# Delirium severity does not differ between medical and surgical intensive care units after adjusting for medication use

**DOI:** 10.1038/s41598-022-18429-9

**Published:** 2022-08-24

**Authors:** Damaris Ortiz, Heidi L. Lindroth, Tyler Braly, Anthony J. Perkins, Sanjay Mohanty, Ashley D. Meagher, Sikandar H. Khan, Malaz A. Boustani, Babar A. Khan

**Affiliations:** 1 Department of Surgery, Indiana University School of Medicine, 545 Barnhill Dr., Emerson Hall, Indianapolis, IN 46202 USA; 2 Sidney & Lois Eskenazi Hospital Smith Level 1 Trauma Center, 720 Eskenazi Ave, Indianapolis, IN 46202 USA; 3 Center of Health Innovation and Implementation Science, Center for Translational Science and Innovation, Indiana University, 410 W. 10th St, Indianapolis, IN 46202 USA; 4 Indiana University Health Methodist Hospital, 1701 N Senate Blvd, Indianapolis, IN 46202 USA; 5 Sidney & Lois Eskenazi Hospital Smith Level 1 Trauma Center, 720 Eskenazi Avenue, 2nd floor Room 431, Indianapolis, IN 46202 USA; 6 Department of Nursing, Mayo Clinic Nursing Research Division, 200 First Street SW, Rochester, MN 55905 USA; 7 Indiana University School of Medicine, Fort Wayne Campus, 2101 East Coliseum Blvd, Fort Wayne, IN 46805 USA; 8 Indiana University Center of Aging Research, Regenstrief Institute, 1101 W. 10th Street, Indianapolis, IN 46202 USA; 9 Division of Pulmonary, Critical Care, Sleep and Occupational Medicine, Department of Medicine, Indiana University School of Medicine, 1120 W. Michigan St., CL 260, Indianapolis, IN 46202 USA

**Keywords:** Physiology, Diseases, Outcomes research

## Abstract

Severe delirium is associated with an increased risk of mortality, institutionalization, and length of stay. Few studies have examined differences in delirium severity between different populations of critically ill patients. The objective of the study was to compare delirium severity and the presence of the four core features between adults in the surgical intensive care unit (SICU) and medical intensive care unit (MICU) while controlling for variables known to be associated with delirium. This is a secondary analysis of two parallel randomized multi-center trials conducted from March 2009 to January 2015 at 3 Indianapolis hospitals. A total of 474 adults with delirium were included in the analysis. Subjects were randomized in a 1:1 ratio in random blocks of 4 by a computer program. Patients were randomized to either haloperidol prescribing or de-prescribing regimen vs usual care. Delirium severity was assessed daily or twice-daily using the CAM-ICU-7 beginning after 24 h of ICU admission and until discharge from the hospital, death, or 30 days after enrollment. Secondary outcomes included hospital length of stay, hospital and 30-day mortality, and delirium-related adverse events. These outcomes were compared between SICU and MICU settings for this secondary analysis. Out of 474 patients, 237 were randomized to intervention. At study enrollment, the overall cohort had a mean age of 59 (SD 16) years old, was 54% female, 44% African-American, and 81% were mechanically ventilated upon enrollment. MICU participants were significantly older and severely ill with a higher premorbid cognitive and physical dysfunction burden. In univariate analysis, SICU participants had significantly higher mean total CAM-ICU-7 scores, corresponding to delirium severity, (4.15 (2.20) vs 3.60 (2.32), p = 0.02), and a lower mean RASS score (− 1.79 (1.28) vs − 1.53 (1.27), p < 0.001) compared to MICU participants. Following adjustment for benzodiazepines and opioids, delirium severity did not significantly differ between groups. The presence of Feature 3, altered level of consciousness, was significantly associated with the SICU participants, identifying as Black, premorbid functional impairment, benzodiazepines, opioids, and dexmedetomidine. In this secondary analysis examining differences in delirium severity between MICU and SICU participants, we did not identify a difference between participant populations following adjustment for administered benzodiazepines and opioids. We did identify that an altered level of consciousness, core feature 3 of delirium, was associated with SICU setting, identifying as Black, activities of daily living, benzodiazepines and opioid medications. These results suggest that sedation practice patterns play a bigger role in delirium severity than the underlying physiologic insult, and expression of core features of delirium may vary based on individual factors.

**Trial registration CT#**: NCT00842608.

## Introduction

Delirium is a type of acute encephalopathy characterized clinically by the presence of four core features; an acute and fluctuating state of consciousness or cognition (feature 1), inattention (feature 2), disturbed level of arousal (feature 3), and disorganized thinking (feature 4)^1^. While present in a variety of healthcare settings^2^, delirium is most prevalent in medical and surgical intensive care units (MICU and SICU, respectively) where up to 80% of older patients are affected^3–12^. Delirium severity is a growing area of study in critical care. The severity of delirium varies between patients with higher levels of delirium severity predictive of longer hospital stays and an increased risk of mortality^12–14^. Despite this, routine assessment of delirium severity is not a current standard of practice in medical or surgical ICU’s.

Medical and surgical ICUs treat distinct patient populations based on diagnosis and potential need for surgical intervention. These distinct patient populations have similar yet different delirium risk profiles prior to hospitalization. Further, the inflammatory response to the precipitating injury is likely different. These differences may alter the presentation and severity of delirium, however, most ICU-related delirium studies to-date combine medical and surgical patients or only evaluate specific populations, such as cardiac or noncardiac surgery patients^8,15–27^. Some surgical literature focuses on pharmacologic delirium prevention or treatment, such as with dexmedetomidine^22,23,25^. Most existing studies describe and categorize delirium, report binary outcomes such as presence or absence of delirium, or investigate risk factors for delirium. Now research is advancing to study the continuum of delirium, including its varied expression and severity. Examining differences in delirium severity, and the presentation of the core features of delirium between MICU and SICU patient populations may identify modifiable practices in critical care leading to the mitigation of delirium severity and its sequelae.

To our knowledge, there are currently no studies comparing delirium severity between MICU and SICU settings. This secondary analysis of data from randomized controlled trials seeks to address this gap in the literature. We hypothesized that the baseline vulnerability of medical patients for delirium would result in more severe delirium in the MICU, and that there would be a significant difference in expression of the core features of delirium between the MICU and SICU patients.

## Methods

This was a secondary data analysis of longitudinal data collected in two federally funded randomized controlled clinical trials; the Pharmacologic Management of Delirium (PMD) in the ICU (CT#: NCT00842608, 3/2009-1/2015) and the parallel study deprescribe-PMD (de-PMD) These trials did not show a benefit of empiric scheduled haloperidol or a medication de-prescribing regimen on delirium onset^12,28^. The Indiana University institutional review board approved the original trials and details were previously published^29^. The patients’ legally authorized representatives provided written informed consent and all research was conducted according to current guidelines.

### Study setting and participants

**Figure 1:**
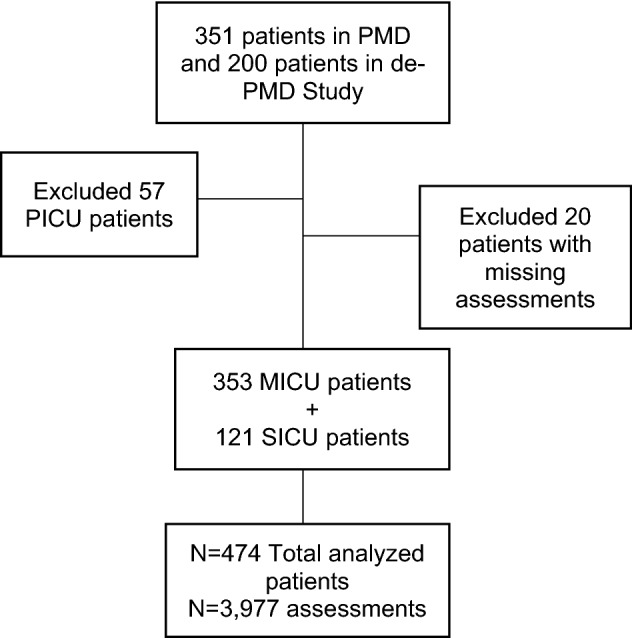
Study participants. PICU, Progressive Intermediate Care Unit; PMD, pharmacological management of delirium; de-PMD, de-prescribing in the pharmacological management of delirium.

The dataset consisted of patients with delirium admitted to the ICU services of three Indianapolis hospitals who were enrolled in either of two trials. This analysis focused on participants assigned to either the MICU or the SICU. Figure 1 describes the patients included in the final analysis. Participant race was obtained from the Electronic Medical Record (EMR) or from the patient’s authorized representative per the study protocols. The categories for race in these studies included Black (n = 210), White (n = 259), Hispanic (n = 2), Asian/Pacific Islander (n = 2), and Other (n = 1) and determined the terminology for this paper. The majority of participants identified as Black or White. Participants were categorized as MICU or SICU per independent chart review by the investigator utilizing the following diagnoses: Respiratory, Respiratory and Sepsis, Sepsis, Altered Mental Status, Neurologic, Gastrointestinal (GI), Trauma, Surgery, and Other (representing other GI and Cardiovascular).

The three hospitals are teaching institutions associated with the Indiana University School of Medicine. Between them there are about 130 ICU beds. Two of the hospitals are level-1 trauma centers with a surgical ICU case mix of primarily trauma, general surgery, orthopedics, neurosurgery, obstetrics and gynecology, and vascular surgery. One hospital is a tertiary care facility with a cancer center. The surgical ICU case mix includes hepatobiliary, thoracic surgery, otolaryngology, urology, and general surgery.

### Inclusion and exclusion criteria

Enrollment criteria for the two trials was previously published^29^. In brief, patients who were 18 years of age or older and had delirium based on the Confusion Assessment Method for the ICU (CAM-ICU)^30,31^ were eligible for study enrollment. Participants who were non-English speaking, hearing impaired, legally blind, admitted with alcohol intoxication, prisoners, had prior history of severe mental illness, stroke, or neurocognitive disorder, had a traumatic brain injury, were pregnant/nursing, or were enrolled in another study were excluded from the study. Informed consent was obtained by the participant’s surrogate decision maker.

### Baseline demographic and clinical variables

Baseline demographics included age, sex, years of education and race. Medical comorbidities were assessed with the Charlson Comorbidity Index (CCI) and illness severity using the Acute Physiology and Chronic Health Evaluation II scale (APACHE II). These were obtained from the electronic medical record (EMR). Baseline cognitive and functional status were obtained by the Informant Questionnaire on Cognitive Decline in the Elderly (IQCODE) and the Instrumental Activities of Daily Living (iADL)^32–34^. This information was provided by the patient’s surrogate decision maker. Data on administered medications (sedatives including propofol, dexmedetomidine, and benzodiazepines, opioids, and antipsychotics including haloperidol, quetiapine, olanzapine, clonidine, and risperdal) were extracted from the EMR. Lorazepam daily dose represents lorazepam equivalents of any benzodiazepine administered. Similarly, morphine milligram equivalent (MME) represents the morphine daily dose equivalent for all opioids administered.

### Primary and secondary outcomes

The primary outcomes were the comparison of delirium severity and the presence of core delirium features between the MICU and SICU utilizing the CAM-ICU-7 delirium severity scale. For every 1 point increase in the scale, the odds of in-hospital mortality increase by 47%, and the odds of discharge home decrease by 20%^35^. The dataset included 1–2× daily assessments of participants’ level of consciousness using the Richmond Agitation and Sedation Scale (RASS), delirium using the Confusion Assessment Method for the ICU (CAM-ICU), and delirium severity using the CAM-ICU-7 from enrollment until death or hospital discharge. The CAM-ICU-7 is an objective 7-point scale (0–7) with high internal consistency (Cronbach alpha of 0.85) for measuring delirium severity. It is based on the four core diagnostic features and scored directly from the CAM-ICU and RASS^35^. Supplemental eTable 1 describes the scoring of the CAM-ICU-7. For this analysis, we used all available patient data from study enrollment through Day 7 of the hospital stay (n = 474 participants, n = 3977 assessments). This time period was chosen due to the increase of participant attrition past Day 7 due to death or discharge. The first study protocols (1/1/2009–07/01/2010, n = 455 assessments) outlined once daily assessments, including coma. This was adjusted to twice daily assessments on July 1st, 2010 (n = 3522 assessments). Participants with a RASS of − 4 or − 5 were labeled as “coma” and were assigned a CAM-ICU-7 score of “7” for this analysis^36–38^.

Secondary outcomes included clinical outcomes, which are defined as inpatient mortality, 30-day mortality, delirium-related adverse event, length of ICU and hospital stays, ventilator days, and discharge disposition. Adverse events were reported to an independent data safety monitoring board (DSMB) throughout the study period.

### Statistical analysis

Demographics, clinical characteristics, delirium severity and core delirium features were summarized using descriptive statistics including mean (standard deviation), median (range) and N (%) based on data type. Differences in these characteristics between MICU and SICU patients were examined using Wilcoxon Rank-Sum test, two sample T-tests, and Fisher’s exact test, dependent on type and distribution of data.

The primary outcomes of the study were to assess for a difference in delirium severity and the presence of core delirium features between the MICU and SICU participants. Univariate analyses were performed followed by multivariable linear regression with delirium severity as the dependent variable. The model included the statistically significant variables from the univariate analysis and variables with clinical significance, including randomization allocation and sedative, opioid, and psychotropic medications by daily dose. The initial regression analysis included coma scored with a CAM-ICU-7 score of 7. To determine delirium feature expression, the four core features of delirium are defined and scored as follows: (1) Acute onset/fluctuating course, 0–1; (2) Inattention, 0–2; (3) Altered level of consciousness, 0–2; and (4) Disorganized thinking, 0–2. Detailed scoring of the CAM-ICU-7 is shown in Supplemental eTable 1. The mean of each feature score over the 7-day assessment window was calculated. An exploratory univariate analysis excluding coma scores was performed to identify which differences between the SICU and MICU participants persisted. (see supplemental eTable 2). Then a linear multivariable regression exploratory analysis was used to determine if the inclusion of coma scores affected variables associated with delirium severity. This exploratory analysis was then repeated with feature 3 (Altered Level of Consciousness) as the dependent variable since it remained significant in the initial exploratory univariate analysis. For these exploratory analyses assessments indicating “coma” with a RASS score of − 4 or − 5 were *not* imputed as a CAM-ICU-7 score of “7”.

We used Fisher’s exact test to determine if mortality, discharge disposition, and presence of an adverse event differed between the MICU and SICU. Wilcoxon Rank Sum test was to compare hospital days, ICU days, and mechanical ventilations days between the MICU and SICU.

### Ethics approval and consent to participate

This study was approved by the Indiana University Institutional Review Board. Informed consent for the study was obtained by all participants’ legal representatives.

## Results

### Descriptive characteristics

A total of 474 patients with delirium severity assessments were included in this analysis. Patients excluded due to missing data or being in the PICU were noted to have a higher Charlson Comorbidity Index, a higher rate of mechanical ventilation, and older age, as seen in supplemental eTable 5. At study enrollment, the overall cohort had a mean age of 59 (Standard Deviation 16) years old, was 54% female, 44% Black, and 81% were mechanically ventilated upon enrollment. Further clinical characteristics are shown in Table 1. MICU participants were significantly older (61 (14) vs 54 (16) years, p < 0.001), more likely to be female (58% vs 43%, p < 0.005), had higher illness severity (APACHEII, 21.28 (8.19) vs 16.77 (7.84), p < 0.001), more comorbidities (CCI, 3.23 (2.72) vs 1.77 (2.41), p < 0.001) and worse baseline cognition (IQCODE, 3.20 (0.47) vs 3.14 (0.27), p = 0.005). The SICU participants had significantly higher baseline functional status (iADL) scores (7.08 (1.93) vs 6.01 (2.57), p < 0.001). Eligibility for haloperidol prescribing was significant between groups with more patients in the SICU eligible to receive the haloperidol intervention (88 (73) vs (210 (59) p < 0.01). Daily doses of benzodiazepines, opioids, and quetiapine were significantly higher in the SICU than in the MICU as shown in Table 2.

**Table 1:** Baseline demographic and clinical characteristics.

Demographic and clinical characteristics	Total sample (n = 474)	SICU (n = 121)	MICU (n = 353)	*P* value
**Mean [SD]**				
Age^a^	59 [16]	54 [19]	61 [14]	< 0.001
Education (years)	11.56 [2.25]	11.53 [2.20]	11.57 [2.27]	0.88
Illness severity (APACHE II)^a^	20.14 [8.34]	16.77 [7.84]	21.27 [8.19]	< 0.001
Charlson Comorbidity Index (CCI)^a^	3.00 [2.74]	1.77 [2.41]	3.42 [2.72]	< 0.001
Premorbid cognition (IQCODE)^a^	3.19 [0.45]	3.14 [0.37]	3.20 [0.47]	0.005
Premorbid function (iADL)^a^	6.29 [2.46]	7.08 [1.93]	6.02 [2.57]	< 0.001
**No. [%] with data**				
Female^a^	256 (54)	52 (43)	204 (58)	0.006
Black	210 (45)	55 (45)	155 (44)	0.83
Mechanical ventilation	385 (81)	100 (83)	285 (81)	0.69
Haloperidol eligible arm^a,b^	298 (63)	88 (73)	210 (59)	0.009
Randomized to intervention^c^	237 (50)	63 (52)	174 (49)	0.67

This table presents the study demographics and clinical characteristics of the overall cohort and in each ICU setting.

*APACHE II* illness severity, *IADL* instrumental activities of daily living (Lawton), *ADL* activities of daily living (Katz), *SICU* surgical intensive care unit, *MICU* medical intensive care unit, *CCI* Charlson Comorbidity Index, *IQCODE* informant questionnaire on cognitive decline in the elderly.

^a^Statistically significant differences between MICU and SICU.

^b^Haloperidol Eligible Arm refers to the patients without contraindications to the haloperidol intervention for the PMD study.

^c^Randomized to Intervention refers to the patients assigned to either haloperidol prescribing arm or the de-prescribing only arm of the PMD and de-PMD studies, respectively, as opposed to usual care.

**Table 2:** Sedative, opioid, and psychotropic medication use.

Medication daily dose in Mg	Total sample (n = 474)	SICU (n = 121)	MICU (n = 353)	*P* value
**Median [IQR]**				
Lorazepam equivalents^a^	0.4 [0.0, 3.70]	1.1 [0.0, 10.50]	0.3 [0.0, 2.00]	0.007
Propofol	0.0 [0.0, 271.60]	0.0 [0.0, 127.5]	0.0 [0.0, 272.4]	0.52
Dexmedetomidine	0.0 [0.0, 0.0]	0.0 [0.0, 0.0]	0.0 [0.0, 0.0]	0.38
Haloperidol	0.0 [0.0, 0.6]	0.0 [0.0, 1.3]	0.0 [0.0, 0.4]	0.06
Quetiapine^a^	0.0 [0.0, 0.0]	0.0 [0.0, 0.0]	0.0 [0.0, 0.0]	< 0.001
Risperdal	0.0 [0.0, 0.0]	0.0 [0.0, 0.0]	0.0 [0.0, 0.0]	0.09
Olanzapine	0.0 [0.0, 0.0]	0.0 [0.0, 0.0]	0.0 [0.0, 0.0]	0.50
Clonidine	0.0 [0.0, 0.0]	0.0 [0.0, 0.0]	0.0 [0.0, 0.0]	0.06
Morphine equivalents^a^	32.6 [3.4, 100.3]	56.7 [5.4, 149.0]	29.2 [2.5, 92]	0.002
**Any medication use in first 7 days**				
No. (%) with data				
Lorazepam equivalents	309 (65)	83 (69)	226 (64)	0.38
Propofol	186 (39)	43 (36)	143 (41)	0.39
Dexmedetomidine	41 (9)	13 (11)	28 (8)	0.35
Haloperidol	170 (36)	48 (40)	122 (35)	0.32
Quetiapine^a^	37 (8)	21 (17)	16 (5)	< 0.001
Risperdal	1 (.2)	1 (1)	0 (0)	0.26
Olanzapine	7 (2)	1 (1)	6 (2)	0.68
Clonidine	24 (5)	10 (8)	14 (4)	0.09
Morphine equivalents	414 (87)	112 (92)	302 (86)	0.06

This table presents doses of sedative, opioid, and psychotropic medications compared between SICU and MICU, expressed as medication daily dose and number of patients who received that medication during the first 7 days.

*SICU* surgical intensive care unit, *MICU* medical intensive care unit, *Mg* milligrams, *IQR* interquartile range, *No.* number.

^a^Statistically significant differences between MICU and SICU.

### Delirium severity, MICU vs SICU

Delirium severity was measured from enrollment until hospital day 7 due to increasing frequency of death (CAM-ICU-7, 3.8 (2.8) or hospital discharge (length of hospital stay, 25.6 (29.8 days) after 7 days in an ICU. In univariate analysis, SICU participants had significantly higher mean total CAM-ICU-7 scores (4.15 (2.20) vs 3.60 (2.32), p = 0.02), and a more negative mean RASS score (− 1.79 (1.28) vs − 1.53 (1.27), p < 0.001) compared to MICU participants (see Table 3). These associations were not maintained following adjustment for benzodiazepines and opioids (see Table 4). The variables that remained associated with delirium severity in multivariable linear regression after accounting for potential confounders in all ICU patients included age, identifying as Black, and daily doses of benzodiazepines and opioid medications. These results remained significant without imputed coma scores.

**Table 3:** Delirium severity, core features, mortality, adverse events, and discharge disposition (including coma).

Clinical values	Total sample (n = 474)	SICU (n = 121)	MICU (n = 353)	*P* value
**Mean [SD]**				
Delirium severity (CAM-ICU-7)^a^	3.74 [2.30]	4.15 [2.20]	3.60 [2.32]	0.02
Acute onset/fluctuation (F1)	0.60 [0.34]	0.65 [0.31]	0.58 [0.35]	0.06
Inattention (F2)^a^	0.99 [0.72]	1.11 [0.71]	0.95 [0.72]	0.03
Altered level of consciousness (F3)^a^	1.14 [0.66]	1.27 [0.61]	1.10 [0.66]	0.01
Disorganized thinking (F4)^a^	1.04 [0.73]	1.17 [0.69]	1.00 [0.74]	0.03
RASS^a^	1.59 [1.28]	− 1.79 (1.28]	− 1.53 [1.27]	0.03
Coma days	1.48 [1.82]	1.69 [1.89]	1.41 [1.79]	0.12
**No. (%) with data**				
ICU mortality	53 (11)	10 (8)	43 (12)	0.32
In-hospital mortality	62 (13)	11 (9)	51 (14)	0.16
30-day mortality	74 (16)	14 (12)	60 (17)	0.20
Adverse events^b^	204 (43)	47 (39)	157 (44)	0.29
Discharge to home	174 (37)	44 (36)	130 (37)	1.00

This table presents the delirium severity, core features, mortality, adverse events and discharge disposition compared between SICU and MICU setting, utilizing imputed coma scores as delirium severity of 7 days.

*CAM-ICU-7* confusion assessment method for intensive care unit-7, *F* feature, *RASS* Richmond agitation and sedation scale.

^a^Statistically significant differences between MICU and SICU.

^b^Delirium-related adverse events included falls, use of physical restraints, injuries such as pulling out IV lines or urinary catheters, re-intubations, and pressure ulcers.

**Table 4:** Regression results for CAM-ICU-7.

	Imputed coma				Coma not imputed			
	Without medications in the model		With medications in the model		Without medications in the model		With medications in the model	
	Estimate (SE)	*P* value	Estimate (SE)	*P* value	Estimate (SE)	*P* value	Estimate (SE)	*P* value
SICU^a^	0.72 (0.26)	0.01	0.38 (0.23)	0.10	0.56 (0.26)	0.03	0.24 (0.24)	0.31
Age^a^	− 0.001 (0.01)	0.94	0.02 (0.01)	0.01	0.003 (0.01)	0.74	0.02 (0.01)	0.02
Female	− 0.22 (0.22)	0.33	− 0.10 (0.19)	0.60	− 0.13 (0.22)	0.55	0.00 (0.20)	0.99
Black^a^	0.77 (0.22)	0.001	0.77 (0.19)	< 0.001	0.54 (0.21)	0.01	0.60 (0.19)	0.002
APACHE	0.00 (0.01)	0.92	0.01 (0.01)	0.51	0.01 (0.01)	0.62	0.01 (0.01)	0.36
CCI	0.06 (0.04)	0.19	0.06 (0.04)	0.13	0.03 (0.04)	0.50	0.04 (0.04)	0.35
IQCODE	− 0.04 (0.30)	0.90	0.15 (0.26)	0.57	0.06 (0.29)	0.83	0.27 (0.27)	0.32
Lawton	− 0.10 (0.05)	0.07	− 0.09 (0.05)	0.07	− 0.08 (0.05)	0.15	− 0.07 (0.05)	0.13
Haloperidol eligible study	0.19 (0.23)	0.40	− 0.01 (0.20)	0.94	0.19 (0.22)	0.39	− 0.02 (0.21)	0.94
Usual care	0.02 (0.22)	0.94	− 0.01 (0.19)	0.96	− 0.14 (0.21)	0.52	− 0.14 (0.19)	0.47
Any quetiapine^a^			0.46 (0.37)	0.21			0.91 (0.37)	0.01
Log daily lorazepam equivalents^a^			0.56 (0.08)	< 0.001			0.39 (0.09)	< 0.001
Log daily MME^a^			0.29 (0.06)	< 0.001			0.28 (0.06)	< 0.001
Any propofol			0.26 (0.21)	0.20			− 0.03 (0.21)	0.90
Any dexmedetomidine			0.70 (0.35)	0.05			0.57 (0.35)	0.10
Log haloperidol dose			0.13 (0.17)	0.45			0.11 (0.18)	0.54

This table presents the linear regression analysis for delirium severity taking into account ICU type as well as other variables including medications. Delirium severity was determined by the mean of the CAM-ICU-7 scores in the first 7 days after enrollment.

*SE* standard error, *APACHE II* acute physiology and chronic health evaluation is a score which measures severity of illness, *IADL* instrumental activities of daily living (Lawton), *ADL* activities of daily living (Katz), *SICU* surgical intensive care unit, *MICU* medical intensive care unit, *CCI* Charlson Comorbidity Index, *IQCODE* informant questionnaire on cognitive decline in the elderly, *MME* morphine milligram equivalent.

^a^Significant differences in regression analysis.

### Delirium core features, MICU vs SICU

SICU participants scored significantly higher on the core delirium features inattention (feature 2), altered level of consciousness (feature 3), and disorganized thinking (feature 4). After removing imputed coma scores, only feature 3, represented by the RASS score, were significantly different between ICU setting (see supplemental eTable 2). In linear regression, feature 3 remained associated with SICU setting, identifying as Black, activities of daily living (Lawton), daily doses of propofol, benzodiazepines and opioids, and any dose of dexmedetomidine. The relationship with propofol was no longer significant after removing coma scores (see supplemental eTable 3).

### Hospital outcomes

SICU participants had longer median hospital and ICU lengths of stay, median [interquartile range] (29 [2–414]) vs (17 [4–114]) days, p < 0.0001, and (22 [15–37.5]) vs (14 [10–23]), p < 0.001), respectively. SICU patients also had more median days on mechanical ventilation compared to MICU patients (4 [0–7]) vs (2 [0–5]), p 0.009. There was no difference in 30-day mortality, discharge disposition, or adverse events. These results are displayed in Table 3 and supplemental eTable 4.

## Discussion

This secondary analysis identified significant demographic and clinical differences between MICU and SICU study participants. We did not identify a difference in delirium severity between these two patient populations following adjustment for benzodiazepines and opioids. Age, identifying as Black, and the daily dose of benzodiazepines and opioids remained significantly associated with delirium severity in the final model. Core feature 3 of delirium, an altered level of consciousness, remained significantly associated with SICU participants after accounting for coma status, medications, age, sex, race, illness severity, comorbidities, and baseline cognition.

Increased delirium severity is associated with increased mortality, longer hospital lengths of stay, and healthcare institutionalization^12–14,39^. We hypothesized that MICU participants would have worse delirium severity, extrapolating from factors associated with delirium onset. Participants in the MICU more frequently have diagnoses of respiratory, infectious disease, and endocrine/metabolic origins. They also have more severe acute illness by the Acute Physiology and Chronic Health Evaluation (APACHE) scores^40^. While the MICU patient population has more predisposing factors for delirium, the SICU population may have more precipitating factors including exposure to anesthesia, surgery, and sedative and hypnotic medications^41–44^. Surprisingly, despite the older age, higher illness severity, decreased functional status and higher baseline cognitive impairment of the MICU cohort, the SICU participants still had higher delirium severity in univariate analysis. However, this distinction disappeared after adjusting for benzodiazepine and opioid medications. Previous studies report an increased likelihood of delirium following administration of benzodiazepines and opioid medications^27,40,41^. Our findings substantiate these relationships. In fact, the administration of deliriogenic medications eliminated the statistical importance of the baseline vulnerability to delirium. These findings further support the imperative to minimize the clinical use of deliriogenic medications^38^.

In this analysis, we examined if MICU and SICU participants differed in their expression of the core features of delirium. When coma was imputed as a CAM-ICU-7 score of 7, indicating severe delirium, SICU participants had a higher prevalence of inattention (feature 2), altered level of consciousness (feature 3), and disorganized thinking (feature 4). When the model was adjusted for coma (imputed values removed), benzodiazepines, and opioids, only feature 3 retained statistical significance with SICU participants. These findings, along with the higher daily doses of sedative medications and more negative RASS scores in the SICU participants, may indicate a persistent practice of heavily sedating critically ill surgical patients, thereby potentially masking the severity of delirium. Procedural sedation and general anesthesia for surgical interventions may also contribute to this observed relationship. Recent studies using the bispectral index (BIS)-guided titration of anesthesia during surgery have shown a reduction in postoperative delirium and 3-month postoperative cognitive decline in elderly patients undergoing major noncardiac surgery^45,46^. Further research is needed to replicate these findings.

Interestingly, identifying as Black was the only demographic or clinical characteristic that significantly correlated with delirium severity and feature 3, in addition to benzodiazepine and opioid medications. No studies to our knowledge have examined the relationships between race and ethnicity and the core features of delirium. In a recently published study from this cohort, identifying as Black was associated with belonging to the *Severe-Slow Recovers* and *Severe-Non-recovers* delirium severity trajectories^47^. However, these findings require further investigation as past studies have reported conflicting results^48^, and none have reported on race and ethnicity associated with delirium severity. There is established literature, however, on the inter-ethnic and inter-racial variability of responses to anesthesia, particularly propofol^49^. Senegalese African Black patients as well as Chinese and Indian patients from Malaysia have been noted to require lower propofol doses for induction and have slower recovery times from general anesthesia compared to Caucasian patients from Italy^50^. These differences, in addition to varying responses to opioids^51,52^, are areas of further research that can help develop individualized sedation and analgesic strategies that are safe and effective^53^.

Despite the inherent differences between patients categorized as “medical” or “surgical,” treatment practices particularly in relation to anesthesia and sedation may have more of an impact on delirium than the underlying illness. Furthermore, race and ethnicity may have an association with delirium severity.

### Strengths and limitations

Our study has several limitations. First, all participants had delirium, therefore we were not able to compare the incidence of delirium between groups. Second, certain information about the surgical ICU patients was not available, including types of surgeries, the American Society of Anesthesiologists (ASA) physical status classifications, and whether patients underwent emergency surgeries. Third, this study data is from 2009 to 2015 when sedation recommendations and practices were evolving. The clinical significance of quetiapine may be negligible due to the infrequent use of antipsychotics in either setting. Similarly, the clinical significance of the differences in activities of daily living (iADL) are negligible. In addition, all study sites were academic hospitals with ongoing research in delirium; therefore, clinical practices may limit the generalizability of our findings. This study focuses on practice patterns in the United States which also may limit generalizability. The clinical trials also had limited categorization of race and ethnicity.

To our knowledge this study is the first to compare delirium severity and the core delirium features between the surgical and medical ICU populations. It is also the first to describe an association between participants admitted to a surgical ICU, delirium severity, core feature 3, and race and ethnicity. These results can help generate additional research questions on the association between sedative medications, race and ethnicity, and delirium severity.

## Conclusions

This secondary analysis did not identify a difference in delirium severity between the medical and surgical ICU population following adjustment for benzodiazepines and opioids. This may indicate that sedation practices have more impact on delirium severity than underlying illness and physiologic insult. Surgical ICU patients may be at higher risk of increased delirium severity due to exposure to more sedation and anesthesia, which are modifiable risk factors. Further study is needed on the effects of sedative medications on patients based on race and ethnicity.

## Supplementary Information

Supplementary Tables.

## Data Availability

Data is available upon request.

## Abbreviations

ICU: Intensive care unit
MICU: Medical intensive care unit
SICU: Surgical intensive care unit
PMD: Pharmacologic management of delirium
De-PMD: Deprescribing in the pharmacologic management of delirium
EMR: Electronic medical record
GI: Gastrointestinal
PICU: Progressive intermediate care unit
CAM-ICU: Confusion assessment method for the ICU
CCI: Charlson Comorbidity Index
APACHE II: Acute physiology and chronic health evaluation II scale
IQCODE: Informant questionnaire on cognitive decline in the elderly
iADL: Instrumental activities of daily living
MME: Morphine milligram equivalent
DSMB: Data safety monitoring board
RASS: Richmond agitation and sedation scale
BIS: Bispectral index
