# Supplementary material for: Delirium severity does not differ between medical and surgical intensive care units after adjusting for medication use
Source: Sci Rep. 2022 Aug 24;12:14447. doi: 10.1038/s41598-022-18429-9 (PMC9402532; doi:10.1038/s41598-022-18429-9)
Supplement: Supplementary file 1 — Supplementary Tables. [file 41598_2022_18429_MOESM1_ESM.docx]

**Supplemental eTable1**. Scoring of Delirium Severity using CAM-ICU-7

| **Table E1: CAM-ICU-7 Scoring System** | | |
| --- | --- | --- |
| **CAM-ICU** | **Answer** | **CAM-ICU-7 Score** |
| **Feature 1 Acute Onset/ Fluctuating Course** | Negative  Positive | 0  1 |
| **Feature 2 Inattention** | All correct  4-7 Correct  0-3 Correct | 0  1  2 |
| **Feature 3 Altered Level of Consciousness** | RASS of 0  RASS -1 to +1  RASS <-1 or >+1 | 0  1  2 |
| **Feature 4 Disorganized Thinking** | All Correct  2-3 Correct  0-1 Correct | 0  1  2 |
| **Total** |  | **0-7** |
| CAM-ICU-7 Scoring:  0 = No delirium symptoms  1-2 = Subsyndromal delirium  3-5 = Mild-moderate delirium  6-7 = Severe delirium | | |

**Supplemental eTable2.** Delirium Severity, Core Feature, Mortality, and Discharge Disposition excluding coma

|  | Total Sample  (n=474) | SICU  (n=121) | MICU  (n=353) | *P* value |
| --- | --- | --- | --- | --- |
| **Mean (SD)** |  |  |  |  |
| Delirium Severity (CAM-ICU7) | 3.07 (2.18) | 3.37 (2.22) | 2.97 (2.16) | .08 |
| Acute Onset/Fluctuation (F1) | .53 (.35) | .56 (.35) | 0.52 (0.35) | .24 |

| Inattention (F2) | .77 (.72) | .84 (.77) | 0.75 (0.70) | .39 |
| --- | --- | --- | --- | --- |

| Altered Level of Consciousness (F3)^a^ | 1.00 (.63) | 1.13 (.61) | 0.96 (0.64) | .01 |
| --- | --- | --- | --- | --- |
| Disorganized Thinking (F4) | .88 (.73) | .98 (.73) | 0.84 (0.73) | 0.08 |
| RASS^a^ | -1.59 (1.28) | -1.79 (1.28) | -1.53 (1.27) | 0.03 |

| Coma (days) | 1.48 (1.82) | 1.69 (1.89) | 1.41 (1.79) | 0.13 |
| --- | --- | --- | --- | --- |

|  |  |  |  |  |
| --- | --- | --- | --- | --- |
| **n (%)** |  |  |  |  |

| 7-day In-hospital Mortality | 53 (11) | 10 (8) | 43 (12) | .32 |
| --- | --- | --- | --- | --- |

| 30-Day Mortality | 74 (16) | 14 (12) | 60 (17) | .19 |
| --- | --- | --- | --- | --- |
| Discharge to Home | 174 (37) | 44 (36) | 130 (37) | 1.00 |

a: significant difference between SICU and MICU

**Supplemental eTable3**. Regression results for feature 3 (Altered Level of Consciousness)

|  | Imputed Coma | | | | Coma Not Imputed | | | |
| --- | --- | --- | --- | --- | --- | --- | --- | --- |
|  | Without Medications in the Model | | With Medications in the Model | | Without Medications in the Model | | With Medications in the Model | |
|  | Estimate (SE) | *P* Value | Estimate (SE) | *P* Value | Estimate (SE) | *P* Value | Estimate (SE) | *P* value |
| SICU^a^ | .24 (.07) | .001 | .17 (.07) | .01 | .25 (.07) | .001 | .19 (.07) | .01 |
| Age | -.002 (.002) | .37 | .003 (.002) | .07 | -.002 (.002) | .33 | .003 (.002) | .19 |
| Female | -.02 (.06) | .79 | .01 (.05) | .91 | -.005 (.06) | .94 | .02 (.06) | .67 |
| Black^a^ | .23 (.06) | <.001 | .22 (.05) | <.001 | .17 (.06) | .005 | .17 (.05) | .002 |
| APACHE | .00 (.00) | .50 | .00 (.00) | .17 | .00 (.00) | .27 | .01 (.00) | .09 |
| CCI | .02 (.01) | .11 | .02 (.01) | .06 | .02 (.01) | .15 | .02 (.01) | .06 |
| IQCODE | -.10 (.09) | .24 | -.06 (.07) | .45 | -.12 (.08) | .17 | -.07 (.08) | .34 |
| Lawton^a^ | -.04 (.02) | .02 | -.03 (.01) | .01 | -.04 (.02) | .01 | -.04 (.01) | .004 |
| Haldol Eligible Study | .06 (.06) | .33 | .00 (.06) | .94 | .09 (.06) | .15 | .03 (.06) | .57 |
| Usual Care | -.03 (.06) | .66 | -.03 (.05) | .57 | -.09 (.06) | .13 | -.09 (.05) | .10 |
| Any Quetiapine |  |  | .03 (.10) | .80 |  |  | .07 (.10) | .52 |
| Log Daily Benzodiazepine Dose^a^ |  |  | .15 (.02) | <.001 |  |  | .13 (.02) | <.001 |
| Log Opioid Dose^a^ |  |  | .08 (.02) | <.001 |  |  | .07 (.02) | <.001 |
| Any Propofol^a^ |  |  | .15 (.06) | .01 |  |  | .10 (.06) | .11 |
| Any Dexmedetomidine^a^ |  |  | .25 (.10) | .01 |  |  | .26 (.10) | .01 |
| Log Haldol Dose |  |  | .05 (.05) | .28 |  |  | .06 (.05) | .25 |

eTable3 presents the linear regression results with feature 3 as the dependent variable, accounting for several confounding variables. Models with and without sedative medications and with and without coma imputed are shown.

1. Significant difference in regression analysis

**Supplemental eTable4.** Additional Clinical Outcomes

| Hospital Outcomes | Total Sample (n=474) | SICU (n=121) | MICU (n=353) | *P* Value |
| --- | --- | --- | --- | --- |
| Median [IQR] |  |  |  |  |
| ICU length of stay in days^a^ | 16 [11-26] | 22 [15-37.5] | 14 [10-23] | <.001 |
| Mechanical Ventilation days^a^ | 2 [0-6] | 4 [0-7] | 2 [0-5] | .009 |
| Hospital length of stay in days^a^ | 19 [12-31] | 29 [17-43] | 17 [12-27] | <.0001 |

eTable4 presents additional hospital outcomes compared between SICU and MICU settings.

^a^Significant difference between ICU setting

**Supplemental eTable5**. Comparison of subjects excluded for missing data or stepdown unit

| **Demographic & Clinical Characteristics** | Total Sample  (n=551) | Include  (n=474) | Exclude  (n=77) | *P* Value |
| --- | --- | --- | --- | --- |
| **Mean (SD)** |  |  |  |  |
| Age^a^ | 60.2 (16.0) | 59.0 (15.8) | 67.7 (15.4) | <.001 |
| Illness Severity (APACHE II) | 20.0 (8.2) | 20.1 (8.3) | 18.9 (7.2) | .21 |
| Charlson Comorbidity Index (CCI)^a^ | 3.2 (2.8) | 3.0 (2.7) | 4.2 (3.0) | <.001 |
| **n (%)** |  |  |  |  |
| Female | 302 (55) | 256 (54) | 46 (60) | .39 |
| African-American | 248 (45) | 210 (45) | 38 (50) | .39 |
| Mechanical Ventilation^a^ | 397 (72) | 385 (81) | 12 (16) | <.001 |
| Haldol Eligible Arm | 351 (64) | 298 (63) | 53 (69) | .37 |
| Randomized to Intervention | 273 (50) | 237 (50) | 36 (47) | .63 |

eTable5 examines potential differences between patients included in the analysis and those excluded for missing data or being in the stepdown unit.

a: significant differences in demographic and clinical characteristics between the analyzed sample and those excluded for missing data or being in the stepdown unit.
